# Compression complexity with ordinal patterns for robust causal inference in irregularly sampled time series

**DOI:** 10.1038/s41598-022-18288-4

**Published:** 2022-08-19

**Authors:** Aditi Kathpalia, Pouya Manshour, Milan Paluš

**Affiliations:** Department of Complex Systems, Institute of Computer Science of the Czech Academy of Sciences, 182 07 Prague, Czech Republic

**Keywords:** Climate sciences, Mathematics and computing

## Abstract

Distinguishing cause from effect is a scientific challenge resisting solutions from mathematics, statistics, information theory and computer science. *Compression-Complexity Causality (CCC)* is a recently proposed *interventional* measure of causality, inspired by Wiener–Granger’s idea. It estimates causality based on change in dynamical compression-complexity (or compressibility) of the effect variable, given the cause variable. CCC works with minimal assumptions on given data and is robust to irregular-sampling, missing-data and finite-length effects. However, it only works for one-dimensional time series. We propose an ordinal pattern symbolization scheme to encode multidimensional patterns into one-dimensional symbolic sequences, and thus introduce the *Permutation CCC (PCCC)*. We demonstrate that PCCC retains all advantages of the original CCC and can be applied to data from multidimensional systems with potentially unobserved variables which can be reconstructed using the embedding theorem. PCCC is tested on numerical simulations and applied to paleoclimate data characterized by irregular and uncertain sampling and limited numbers of samples.

## Introduction

Unraveling systems’ dynamics from the analysis of observed data is one of the fundamental goals of many areas of natural and social sciences. In this respect, detecting the direction of interactions or inferring causal relationships among observables is of particular importance that can improve our ability to better understand the underlying dynamics and to predict or even control such complex systems^1,2^.

Around sixty years after the pioneering work of Wiener and Granger^3,4^ on quantifying linear ‘causality’ from observations, it has been widely applied not only in economics^5–7^, for which it was first introduced, but also in various fields of natural sciences, from neurosciences^8^ to Earth sciences^9–11^. A number of attempts have been made to generalize Granger Causality (GC) to nonlinear cases, using, e.g., an estimator based on correlation integral^6^, a non-parametric regression approach^12^, local linear predictors^13^, mutual nearest neighbors^14,15^, kernel estimators^16^, to state a few. Several other causality methods based on the GC principle such as Partial Directed Coherence^17^, Direct Transfer Function^18^ and Modified Direct Transfer Function^19^ have also been proposed.

Information theory has proved itself as a powerful approach into causal inference. In this respect, Schreiber proposed a method for measuring information transfer among observables^20^, known as *Transfer Entropy* (TE), which is based on Kullback-Leibler distance between transition probabilities. Paluš et al.^21^ introduced a causality measure based on mutual information, called *Conditional Mutual Information* (CMI). CMI has been shown to be equivalent to TE^22^. These tools have been applied in various research studies and have shown their power in extracting causal relationships between different systems^23–27^.

We usually work with time series *x*(*t*) and *y*(*t*) as realizations of *m* and *n* dimensional dynamical systems, *X*(*t*) and *Y*(*t*) respectively, evolving in measurable spaces. It means that *x*(*t*) and *y*(*t*) can be considered as the components of these *m* and *n* dimensional vectors. In many cases, only one possible dimension of the phase space is observable, recordings or knowledge of variables which may have indirect effects or play as mediators in the causal interactions between observables may not be available. In this respect, phase-space reconstruction is a common useful approach introduced by Takens^28^, which reconstructs the dynamics of the entire system (including other unknown/unmeasurable variables) using time-delay embedding vectors, as follows: the manifold of an *m* dimensional state vector *X* can be reconstructed as $X(t)=\{x(t),x(t-\eta ),...,x(t-(m-1)\eta )\}$. Here, $\eta$ is the embedding delay, and can be obtained using the embedding construction procedure based on the first minimum of the mutual information^29^. Some causality estimators have applied this phase-space reconstruction procedure to improve their causal inference power, such as high dimension CMI^26^ and TE^30^. Other causality measures, such as, Convergent Cross Mapping (CCM)^31^, Topological Causality^32^, Predictability Improvement^33^, are based directly on the reconstruction of dynamical systems.

Vast amounts of data available in the recent years have pushed some of the above discussed GC extensions, information and phase-space reconstruction based approaches forward as they rely on joint probability density estimations, stationarity, markovianity, topological or linear modeling. However, still, many temporal observations made in various domains such as climatology^34,35^, finance^36,37^ and sociology^38^ are often short in length, have missing samples or are irregularly sampled. A significant challenge arises when we attempt to apply causality measures in such situations^11^. For instance, CMI or TE fail when applied to time series which are undersampled or have missing samples^39–41^ and also in case of time series with short lengths^41^. CCM and kernel based non-linear GC also show poor performance even in the case of few missing samples in bivariate simulated data^42^.

Kathpalia and Nagaraj recently introduced a causality measure, called Compression-Complexity Causality (CCC), which employs ‘complexity’ estimated using lossless data-compression algorithms for the purpose of causality estimation. It has been shown to have the strength to work well in case of missing samples in data for bivariate systems of coupled autoregressive and tent map processes. This has been shown to be the case for samples which are missing in the two coupled time series either in a synchronous or asynchronous manner^41^. Also, it gives good performance for time series with short lengths^41,42^. These strengths of CCC arise from its formulation as an *interventional* causality measure based on the evolution of dynamical patterns in time series, independence from joint probability density functions, making minimal assumptions on the data and use of lossless compression based complexity approaches which in turn show robust performance on short and noisy time series^41,43^. However, as discussed in Ref.^42^, a direct multidimensional extension of CCC is not as straightforward and so a measure of *effective CCC* has been formulated and used on multidimensional systems of coupled autoregressive processes with limited number of variables.

On the other hand, a method for symbolization of phase-space reconstructed (embedded) processes has been used to improve the ability of info-theoretic causality measures for noisy data, such as *symbolic transfer entropy*^44,45^, *partial symbolic transfer entropy*^46,47^, *permutation conditional mutual information* (PCMI)^48^ and *multidimensional PCMI*^49^. The symbolization technique used in these works is based on the Bandt and Pompe scheme for estimation of Permutation Entropy^50^, and often referred to as *permutation* or *ordinal patterns* coding. The scheme labels the embedded values of time-series at each time point in ascending order of their magnitude. Symbols are then assigned at each time point depending on the ordering of values (or the labelling sequence) at that point. Ordinal patterns have been used extensively in the analysis and prediction of chaotic dynamical systems and also shown to be robust in applications to real world time series. By construction, this technique ignores the amplitude information and thus decreases the effect of high fluctuations in data on the obtained causal inference^51^. Other benefits of permutation patterns are: they naturally emerge from the time series and so the method is almost parameter-free; are invariant to monotonic transformations of the values; keep account of the causal order of temporal values and the procedure is computationally inexpensive^52–55^. Ordinal partition has been shown to have the generating property under specific conditions, implying topological conjugacy between phase space of dynamical systems and their ordinal symbolic dynamics^56^. Further, permutation entropy for certain sets of systems has been shown to have a theoretical relationship to the system’s Lyapunov exponents and Kolmogorov Sinai Entropy^57,58^. Because of all these beneficial properties of permutation patterns, it is no wonder that the development of symbolic TE or PCMI helped to make them more robust, giving better performance in the case of noisy measurements, simplifying the process of parameter selection and making less demands on the data.

In this work, we propose the use of CCC approach with reconstructed dynamical systems which are symbolized using ordinal patterns. The combination of strengths of CCC and ordinal patterns, not only makes CCC applicable to dynamical systems with multidimensional variables, but we also observe that the proposed *Permutation CCC* (PCCC) measure gives great performance on datasets with very short lengths and high levels of missing samples. The performance of PCCC is compared with that of PCMI (which is identical to symbolic TE), bivariate CCC and CMI on simulated dynamical systems data. PCCC outperforms the existing approaches and its estimates are found to be robust for short length time series, and high levels of missing data points.

This development for the first time opens up avenues for the use of causality estimation tool on real world datasets from climate and paleoclimate science, finance and other fields where there is prevalence of data with irregular and/or uncertain sampling times. To determine the major drivers of climate is the need of the hour as climate change poses a big challenge to humankind and our planet Earth^59^. Different studies have employed either correlation/coherence, causality methods or modelling approaches to study the interaction between climatic processes. The results produced by different studies are different and sometimes contradictory, presenting an ambiguous situation. We apply PCCC to analyse the causal relationship between the following sets of climatic processes: greenhouse gas concentrations—atmospheric temperature, El-Niño Southern Oscillation—South Asian monsoon and North Atlantic Oscillation—European temperatures at different time-scales and compare its performance with bivariate CCC, bivariate and multidimensional CMI, and PCMI. The time series available for most of these processes are short in length and sometimes have missing samples and (or) are sampled in irregular intervals of time. We expect our estimates to be reliable and to be helpful to resolve the ambiguity presented by existing studies.

## Results

### Simulation experiments

Time series data from a pair of unidirectionally coupled Rössler systems were generated as per the following equations:

1 $$\begin{aligned} \dot{x}_{1}&= - \omega _{1} y_{1} - z_{1}, \nonumber \\ \dot{y}_{1} & = \omega _{1} x_{1} + a_1 \; y_{1}, \nonumber \\ \dot{z}_{1} & = b_1 + z_{1} (x_{1} - c_1), \end{aligned}$$

for the autonomous or master system, and

2 $$\begin{aligned} \dot{x}_{2} & = - \omega _{2} y_{2} - z_{2} + \epsilon (x_{1} - x_{2}), \nonumber \\ \dot{y}_{2}&= \omega _{2} x_{2} + a_2 \; y_{2}, \nonumber \\ \dot{z}_{2}&= b_2 + z_{2} (x_{2} - c_2), \end{aligned}$$

for the response or slave system. Parameters were set as: $a_1 = a_2 = 0.15$, $b_1 = b_2 = 0.2$, $c_1 = c_2 = 10.0$, and frequencies set as: $\omega _{1} = 1.015$ and $\omega _{2} = 0.985$. The coupling parameter, $\epsilon$, was fixed to 0.09. The data were generated by numerical integration based on the adaptive Bulirsch–Stoer method^60^ using a sampling interval of 0.314 for both the master and slave systems. This procedure gives 17–21 samples per one period. 100 realizations of these systems were simulated and initial 5000 transients were removed before using the data for testing experiments.

As can be seen from the equations, there is a coupling between $x_1$ and $x_2$, with $x_1$ influencing $x_2$. The analysis of the causal influence between the two systems was done using the causality estimation measures: bivariate or scalar CCC, CMI, PCCC and PCMI for the cases outlined in the following paragraphs. The estimation procedure for each of the methods is described in the “Methods” section. The values of parameters used for each of the methods are also given in the “Methods” section (Table 2).

#### Finite length data

**Figure 1:**
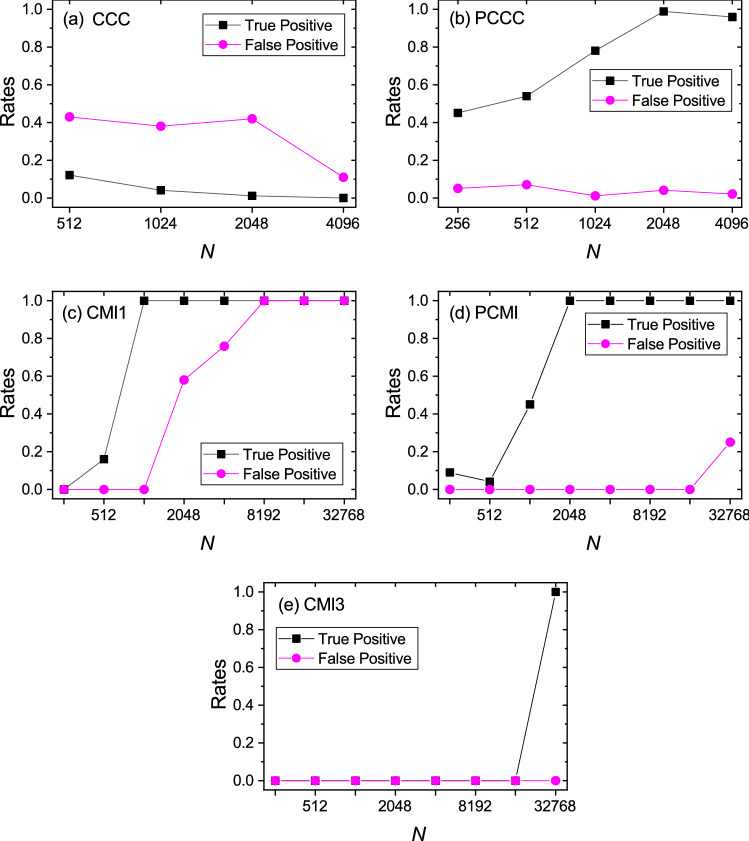
Specificity and sensitivity of methods with varying length. True positive rate (or rate of significant causality estimated from $x_1 \rightarrow x_2$) and false positive rate (or rate of significant causality estimated from $x_2 \rightarrow x_1$), using measures (**a**) scalar CCC (CCC), (**b**) permutation CCC (PCCC), (**c**) scalar CMI (CMI1), (**d**) permutation CMI (PCMI) and (**e**) three-dimensional CMI (CMI3), as the length of time series, *N*, is varied.

The length of time series, *N*, of $x_1$ and $x_2$ taken from coupled Rössler systems was varied as shown in Fig. 1. The estimation for CMI and PCMI is done up to a higher value of length as CMI did not give optimal performance until the length became 32,768 samples. Figure 1c shows scalar (simple bivariate) CMI or one-dimensional CMI (CMI1) between $x_1$ and $x_2$ (see Paluš and Vejmelka^22^). This method has high sensitivity but suffers from low specificity. This problem is solved by using conditional CMI or three-dimensional CMI (CMI3), where the information from other variables ($y_1, z_1, y_2, z_2$) is incorporated in the estimation. Its performance is depicted in Fig. 1e. However, it requires larger length of time series for optimal performance. Figure 1a shows the performance of scalar (or simple bivariate) CCC, which is equivalent to the CMI1 case, considering dimensionality. Figure 1b, d show the performance of PCCC and PCMI respectively. For each length level, all 100 realizations of coupled systems were considered and 100 surrogates generated for each realization in order to perform significance analysis of causality estimated (in both directions) from each realization of coupled processes. These surrogates were generated for both the processes using the Amplitude Adjusted Fourier Transform method^61^ and significance testing done using a standard one-sided z-test with p-value set to 0.05 (this was justified as the distributions of surrogates for CCC and CMI methods implemented were found to be Gaussian). Based on this significance analysis, true positive rate (TPR) and false positive rate (FPR) were computed at each length level. A true positive is counted for a particular realization of coupled systems when causality estimated from $x_1$ to $x_2$ is found to be significant and a false positive is counted when causality estimated from $x_2$ to $x_1$ is found to be significant.

As it can be seen from the plots, direct application of scalar CCC completely fails on multidimensional dynamical systems data, yielding low true positives and high false positives. Hence the method displays poor sensitivity as well as specificity. CMI1 also shows poor performance, yielding high false positives. CMI3, which is appropriate to be applied for multi-dimensional data, only begins to give good performance when the length of time series is taken to be greater than 32,768 samples. On the other hand, PCCC begins to give high true positives and low false positives, as the length of time series is increased to 1024 time points, with TPR and FPR reaching almost 1 and 0 respectively as length is increased to 2048 time points. The use of permutation patterns also improves the performance of CMI3 for short length data as it can be seen that PCMI begins to show a TPR of 1 and FPR of 0 for length of time series equal to 2048 time points.

We did further experiments with simulated Rössler data by varying the amount of noise and missing samples in the data. For these cases, performance of PCCC and PCMI alone were evaluated because it can be seen from the ‘varying length’ experiments that scalar CCC and CMI1 do not work for multidimensional dynamical systems data and CMI3 does not perform well for short length data.

#### Noisy data

**Figure 2:**
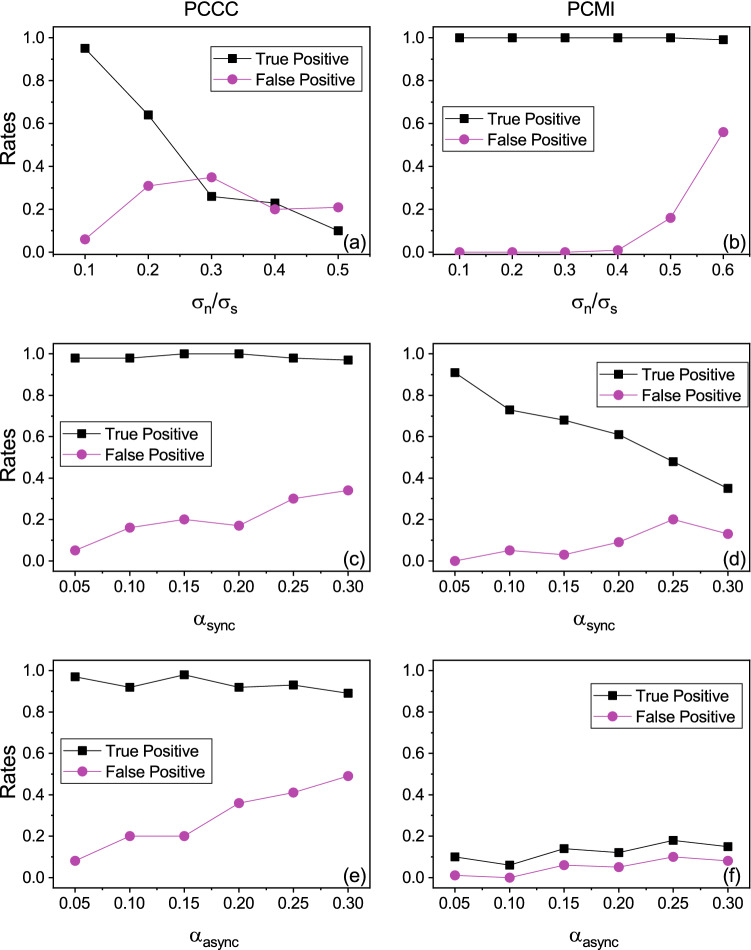
Specificity and sensitivity of methods with varying noise and sparsity. True positive rate (or rate of significant causality estimated from $x_1 \rightarrow x_2$) and false positive rate (or rate of significant causality estimated from $x_2 \rightarrow x_1$), using measures permutation CCC (PCCC) (left column) and permutation CMI (PCMI) (right column) as the level of noise: (**a**, **b**); level of synchronous sparsity: (**c**, **d**); and asynchronous sparsity: (**e**, **f**), are varied.

White Gaussian noise was added to the simulated Rössler data. The amount of noise added to the data was relative to the standard deviation of the data. The noise standard deviation ($\sigma _n$), is expressed as a percentage of the standard deviation of the original data ($\sigma _s$). For example, $20\%$ of noise means $\sigma _n=0.2\sigma _s$, and 100% of noise means $\sigma _n=\sigma _s$. The length of time series taken for this experiment was fixed to 2048. For each realization of noisy data as well, 100 surrogate time series were generated and significance testing performed as before using the Amplitude Adjusted Fourier Transform method and z-test respectively. Figure 2a,b show the results for varying noise in the data for the measures PCCC and PCMI respectively.

It can be seen that PCCC performs well for low levels of noise, up to $10\%$, but at higher levels of noise, its performance begins to deteriorate. PCMI, on the other hand, shows high TPR and low FPR even as the noise level is increased to 50%.

#### Sparse data

We refer to time-series with missing samples as sparse data. Sparsity or non-uniformly missing samples were introduced in the data in two ways: (1) Synchronous sparsity and (2) Asynchronous sparsity. In case of (1), samples were missing from both $x_1$ and $x_2$ at randomly chosen time indices and this set of time indices was the same for both $x_1$ and $x_2$. In case of (2), samples were missing from both $x_1$ and $x_2$ based on two different sets of randomly chosen time indices, that is, the time indices of missing samples were different for $x_1$ and $x_2$. The amount of synchronous/ asynchronous sparsity is expressed in terms of percentage of missing samples relative to the original length of time series taken. $\alpha _{sync}$ and $\alpha _{async}$ refer to the level of missing samples for the cases of synchronous and asynchronous sparsity respectively, and are given by *m*/*N*, where *m* is the number of missing samples and *N* is the original length of time series. *N* was fixed to 2048. The length of time series became shorter as the percentage of missing samples were increased. Causality estimation measures were applied to the data without any knowledge of whether any samples were missing or the time stamps at which the samples were missing. Surrogate data generation for each realization in this case was not done post the introduction of missing samples but prior to that, using the original length time series. Sparsity was then introduced in the surrogate time series in a manner similar to that for original time series.

Figure 2c,d show the results obtained using PCCC and PCMI respectively for synchronous sparsity. Figure 2e,f show the same for asynchronous sparsity. It can be seen that PCCC is robust to the introduction of missing samples, showing high TPR and low FPR. FPR begins to be greater than 0.2 only when the level of synchronous sparsity is increased to $25\%$ and asynchronous sparsity is increased to 20%. PCMI is robust to low levels of synchronous sparsity but deteriorates beyond 5% of missing samples, giving low true positives. It performs very poorly even with low levels of asynchronous sparsity.

### Real data analysis

As discussed in the Introduction, a number of climate datasets are either sampled at irregular intervals, have missing samples, are sampled after long intervals of time or have a combination of two or more of these issues. In addition, their temporal recordings available are short in length. We apply the proposed method, PCCC, to some such datasets described below. We also compare the results obtained with existing measures: scalar CCC, scalar CMI and PCMI.

#### Millenial scale CO$_{2}$-temperature recordings

Mills et al. have compiled independent estimates of global average surface temperature and atmospheric CO$_{2}$ concentration for the Phanerozoic eon. These paleoclimate proxy records span the last 424 million years^62^ and have been used and made available in the study by Wong et al.^63^. One data point for both CO$_{2}$ and temperature recordings were available for each million year period and was used in our analysis to check for causal interaction between between the two.

#### CO$_{2}$, CH$_{4}$ and temperature recordings over the last 800,000 years

Past Interglacials Working Group of PAGES^64^ has made available proxy records of atmospheric CO$_{2}$, CH_4_ and deepwater temperatures over the last 800 ka (1 ka= 1000 years). Each of these time series were reconstructed by separate studies and so the recordings available are non-synchronous and also irregularly sampled for each variable. Further, some data points are missing in the temperature time-series. Roughly, single data point is available for each ka for each of the three variables. CO$_{2}$ proxy data are based on antarctic ice core composites. This was first reported by Lüthi et al.^65^ and the revised values made available in a study by Bereiter et al.^66^. Reconstructed atmospheric CH_4_ concentrations, also based on ice cores, were as reported by Loulergue et al.^67^ (on the AICC2012 age scale^68^). Deepwater temperature recordings obtained using shallow-infaunal benthic foraminifera (Mg/Ca ratios) that became available from Ocean Drilling Program (ODP) site 1123 on the Chatham Rise, east of New Zealand were reported by Elderfield et al.^69^.

Causal influence was checked between CO$_{2}$-temperature and separately between CH_4_-temperature. CO$_{2}$ and CH_4_ data are taken beginning from the 6.5th ka on the AICC2012 scale and temperature data are taken beginning from the 7th ka. Since the number of data points available for temperature are 792, CO$_{2}$-temperature analysis was done based on these 792 samples and as the number of samples of CH_4_ is limited to 756 beginning from the 6.5th ka, CH_4_-temperature analysis was done using these 756 data points.

#### Monthly CO$_{2}$-temperature dataset

Monthly mean CO$_{2}$ data constructed from mean daily CO$_{2}$ values as well as Northern Hemisphere’s combined land and ocean temperature anomalies for the monthly timescale are available open source on the National Oceanic and Atmospheric Administration (NOAA) website. The CO$_{2}$ measurements were made at the Mauna Loa Observatory, Hawaii. A part of the CO$_{2}$ dataset (March 1958–April 1974) were originally obtained by C. David Keeling of the Scripps Institution of Oceanography and are available on the Scripps website. NOAA started its own CO$_{2}$ measurements starting May 1974. The temperature anomaly dataset is constructed from the Global Historical Climatology Network-Monthly data set^70^ and International Comprehensive Ocean-Atmosphere Data Set, also available on the NOAA website. These data from March, 1958 to June 2021 (with 760 data points) were used to check for the causal influence between CO$_{2}$ and temperature on the recent timescale. Both time series were differenced using consecutive values as they were highly non-stationary.

#### Yearly ENSO-SASM dataset

1100 Year El Niño/Southern Oscillation (ENSO) Index Reconstruction dataset, made available open source on NOAA website and originally published in Ref.^71^ was used in this study. South Asian Summer Monsoon (SASM) Index 1100 Year Reconstruction dataset, also available open source on the NOAA website and originally published in Ref.^72^, was the second variable used here. The aim of our study was to check the causal dependence between these two sets of recordings taken from the year 900 AD to 2000 AD (with one data point being available for each year).

#### Monthly NINO-Indian monsoon dataset

Monthly NINO 3.4 SST Index recordings from the year 1870 to 2021 are available open source on the NOAA website. Its details are published in Ref.^73^. All India monthly rainfall dataset from 1871 to 2016, available on the official website of World Meteorological Organization and originally acquired from ‘Indian Institute of Tropical Meteorology’, was used for analysis. These recordings are in the units of mm/month. Causal influence was checked between these two recordings using 1752 data points, ranging from the month January, 1871 to December, 2016.

#### Monthly NAO-temperature recordings

Reconstructed monthly North Atlantic Oscillation (NAO) index recordings from December 1658 to July 2001 are available open source on the NOAA website. The reconstructions from December 1658 to November 1900 are taken from Refs.^74,75^ and from December 1900 to July 2001 are derived from Ref.^76^. Central European 500 year temperature reconstruction dataset, beginning from 1500 AD, is made available open source by NOAA National Centers for Environmental Information, under the World Data Service for Paleoclimatology. These were derived in the study^77^. We took winter only data points (months December, January and February) starting from the December of 1658 to the February of 2001 as it is known that the NAO influence is strongest in winter. This yielded a total of 1029 data points. However, reconstruction based on embedding was done for each year’s winter separately (with a time delay of 1) and not in a continuous manner as for the other datasets, reducing the length of ordinal patterns encoded sequence to 343. Causal influence was checked between NAO and temperature for the encoded sequences using PCMI and PCCC and directly using one-dimensional CMI and CCC for the 1029 length sequences.

#### Daily NAO-temperature recordings

Daily NAO records are available on the NOAA website and have been published in Refs.^78–80^. Daily mean surface air temperature data from the Frankfurt station in Germany were taken from the records made available online by the ECA &D project^81^. This data was taken from 1st January 1950 to 31st April 2021. Once again, daily values from the winter months alone (December, January and February), comprising of 6390 data points, were extracted for the analysis. While embedding the two time series, care was taken not to embed the recordings of winter from one year along with that of winter from the next year. Causal influence was checked between daily winter NAO and temperature time-series.

**Figure 3:**
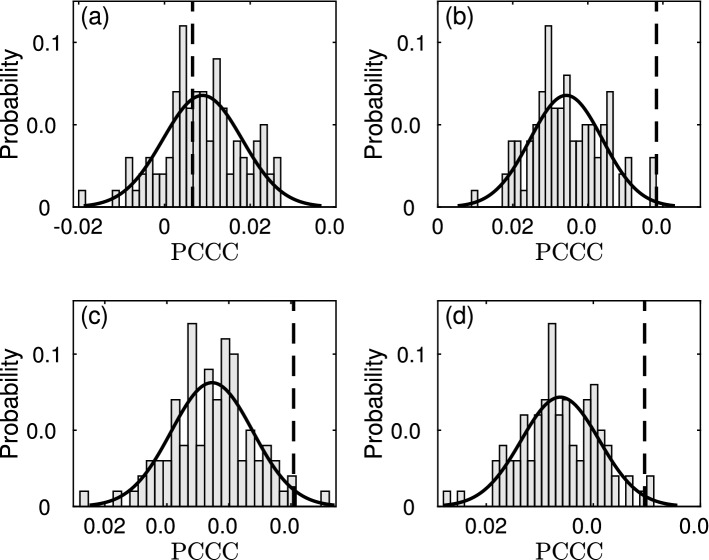
PCCC surrogate analysis results. PCCC surrogate analysis results for: (**a**) Kilo-year scale CO$_{2}$ $\rightarrow$ T, (**b**) Kilo-year scale T $\rightarrow$ CO$_{2}$, (**c**) Yearly ENSO $\rightarrow$ SASM, (**d**) SASM $\rightarrow$ ENSO. Dashed line indicates PCCC value obtained for original series. Its position is indicated with respect to Gaussian curve fitted normalized histogram of surrogate PCCC values. PCCC for cases (**b**)–(**d**) is found to be significant.

For the analysis of causal interaction in each of these datasets, scalar CCC and CMI as well as PCCC and PCMI were computed as discussed in the “Methods” section. Parameters used for each of the methods are also given in the “Methods” section (Table 2). In order to assess the significance of causality value estimated using each measure, 100 surrogate realizations were generated using the *stationary bootstrap* method^82^ for both the time series under consideration. Resampling of blocks of observations of random length from the original time series is done for obtaining surrogate time series using this method. The length of each block has a geometric distribution. The probability parameter that determines the geometric probability distribution for length of each block was set to 0.1 (as suggested in Ref.^82^). Significance testing of the causal interaction between original time-series was then done using a standard one-sided z-test, with p-value set to 0.05. Table 1 shows whether causal influence between the considered variables was found to be significant using each of the causality measures. Figure 3 depicts the value of the PCCC between original pair of time series with respect to the distribution of PCCC obtained using surrogate time series for two datasets: kilo-year scale CO$_{2}$-temperature (Fig. 3a,b) and yearly scale ENSO-SASM (Fig. 3c,d) recordings. In the tables, Fig. 3 and in the following text, we use the notation ‘T’ to refer to temperature generically. Which of the temperature recordings is being referred to, will be clear from context.

**Table 1:** Causal inference obtained for real datasets using different causality measures.

System	Measure				
	Direction	CCC	PCCC	CMI	PCMI
Millenial scale CO$_{2}$ -T	CO$_{2}$ $\rightarrow$ T	$\checkmark$	$\times$	$\times$	$\times$
	T $\rightarrow$ CO$_{2}$	$\checkmark$	$\checkmark$	$\times$	$\times$
Kilo-year scale CO$_{2}$ -T	CO$_{2}$ $\rightarrow$ T	$\times$	$\times$	$\times$	$\times$
	T $\rightarrow$ CO$_{2}$	$\times$	$\checkmark$	$\times$	$\times$
Kilo-year scale CH_4_-T	*CH*_4_ $\rightarrow$ *T*	$\times$	$\checkmark$	$\times$	$\times$
	T $\rightarrow$ CH_4_	$\times$	$\times$	$\times$	$\times$
Monthly scale CO$_{2}$ -T	CO$_{2}$ $\rightarrow$ T	$\times$	$\checkmark$	$\times$	$\times$
	T $\rightarrow$ CO$_{2}$	$\times$	$\times$	$\times$	$\times$
Yearly ENSO-SASM	ENSO $\rightarrow$ SASM	$\times$	$\checkmark$	$\times$	$\times$
	SASM $\rightarrow$ ENSO	$\checkmark$	$\checkmark$	$\times$	$\times$
Monthly NINO-Indian monsoon	NINO $\rightarrow$ Monsoon	$\times$	$\checkmark$	$\checkmark$	$\checkmark$
	Monsoon $\rightarrow$ NINO	$\checkmark$	$\times$	$\checkmark$	$\checkmark$
Monthly NAO-European T	NAO $\rightarrow$ T	$\checkmark$	$\checkmark$	$\times$	$\times$
	T $\rightarrow$ NAO	$\times$	$\times$	$\times$	$\times$
Daily NAO-Frankfurt T	NAO $\rightarrow$ T	$\checkmark$	$\times$	$\checkmark$	$\times$
	T $\rightarrow$ NAO	$\times$	$\times$	$\times$	$\times$

$\checkmark$ indicates significant causality and $\times$ indicates non-significant causality.

## Discussion and conclusions

CCC has been proposed as an ‘interventional’ causality measure for time series. It does not require cause-effect separability in time series samples and is based on dynamical evolution of processes, making it suitable for subsampled time series, time series in which cause and effect are acquired at slightly different spatio-temporal scales than the scales at which they naturally occur and even when there are slight discrepancies in spatio-temporal scales of the cause and effect time series. This results in its robust performance in the case of missing samples, non-uniformly sampled, decimated and short length data^41^. In this work, we have proposed the use of CCC in combination with ordinal pattern encoding. The latter preserves the dynamics of the systems of observed variables, allowing for CCC to decipher causal relationships between variables of multi-dimensional systems while conditioning for the presence of other variables in these systems which might be unknown or unobserved.

Simulations of coupled Rössler systems illustrate how scalar CCC is a complete failure for observables of coupled multi-dimensional dynamical systems, while PCCC performs well to determine the correct direction of coupling. Comparison of PCCC with PCMI for these simulations shows that the former beats the latter by showing better performance on shorter lengths of time series. Further, while PCMI consistently gave superior performance for increasing noise in coupled Rössler systems, experiments with sparse data showed that PCCC outperforms PCMI. This was the case when samples were missing from the driver and response time series either in a synchronous or asynchronous manner.

As PCCC showed promising results for simulations with high levels of missing samples and short length, we have applied it to make causal inferences in datasets from climatology and paleoclimatology which suffer from the issues of irregular sampling, missing samples and (or) have limited number of data points available. Many of these datasets have been analyzed in previous studies. However, different studies report different results probably due to the challenging nature of their recordings available or the limitation of the inference methods applied to work on the data.

For example, the relationship between CO$_{2}$ concentrations and temperature of the atmosphere has been studied from the mid 1800s^83,84^, beginning when a strong link between the two was recognized. Relatively recently, with causal inference tools available, a number of studies have begun to look at the directionality of relationship between the two on different temporal scales. To mention a few findings, Kodra et al.^85^ found that CO$_{2}$ Granger causes temperature. Their analysis was based on data taken from 1860 to 2008. Atanassio^86^ found a clear evidence of GC from CO$_{2}$ to temperature using lag-augmented Wald test, for a similar time range. On the other hand, Stern and Kaufmann^87^ found bidirectional GC between the two, again for a similar time range. Kang and Larsson^88^ also find bidirectional causation between the two using GC, however, by using data from ice cores for the last 800,000 years. Many of these latter studies criticize the former. Also, the drawbacks of one or more of these studies are explicitly mentioned in Refs.^87,89,90^ and highlight the issues with the data and/ or the methodology employed. Other than GC and its extensions, a couple of other measures have also been used to study CO$_{2}$-T relationship. Stips et al.^91^ have applied a measure called Liang’s Information flow on CO$_{2}$-T recordings, both on recent (1850–2005) and paleoclimate (800 ka ice core reconstructions) time-scales. The study finds unidirectional causation from CO$_{2}$ $\rightarrow$ T on the recent time-scale and from T $\rightarrow$ CO$_{2}$ on the paleoclimatic scale. They have also analysed the CH_4_-T relationship and found T to drive CH_4_ on the paleoclimate scale. This study has been criticized by Goulet et al.^92^. They show that an assumption of ‘linearity’ made by Liang’s information flow is nearly always rejected by the data. Convergent cross mapping, which is applied to the 800 ka recordings in another study, finds a bidirectional causal influence between both CO$_{2}$ - T and CH_4_-T^93^. Another recent study, that infers causation using lagged cross-correlations between monthly CO$_{2}$ and temperature, taken from the period 1980–2019, has found a bidirectional relationship on the recent monthly scale, with the dominant influence being from T $\rightarrow$ CO$_{2}$^94^. In the light of the limitations of CCM^95,96^, especially for irregularly sampled or missing data^42^, and of the widely known pitfalls of correlation coefficient^97^, it is difficult to rely on the inferences of the latter two studies.

PCCC indicates unidirectional causality from T $\rightarrow$ CO$_{2}$ on the paleoclimatic scale, using both millenial and kilo-year scale recordings. On the recent monthly scale, the situation is reversed with CO$_{2}$ driving T. These results are in line with some of the existing CO$_{2}$-T causal analysis studies and clearly PCCC does not suffer the limitations of existing approaches. On the kilo-year scale, PCCC suggests that CH_4_ drives T. While none of the above discussed causality studies have found this result, other works have suggested that methane concentrations modulate millenial-scale climate variability because of the sensitivity of methane to insolation^98,99^. Other approaches implemented in this study – CCC, CMI, PCMI also do not duplicate the results obtained by PCCC because of their specific limitations such as the inability to work on multi-dimensional, short length or irregularly sampled data.

ENSO events and the Indian monsoon are other major climatic processes of global importance^59^. The relationship between the two has been studied extensively, especially using correlation and coherence approaches^100–105^. While ENSO is normally expected to play a driving role, there is no clear consensus on the directionality of the relationship between the two processes. More recently, causal inference approaches have been used to study the nature of their coupling. In Refs.^106,107^, both linear and non-linear GC versions were implemented on monthly mean ENSO-Indian monsoon time series, ranging from the period 1871–2006 and bidirectional coupling was inferred between the two processes. Other studies have studied the causal relationship indirectly by analyzing the ENSO-Indian Ocean Dipole link. For example, in Ref.^108^, this connection was studied by applying GC on yearly reanalysis as well as model data ranging from 1950–2014. The study found robust causal influence of Indian Ocean Dipole on ENSO while the influence in opposite direction had lower confidence. Using PCCC, we find a bidirectional causal influence between yearly recordings of ENSO-SASM. However, on the shorter monthly scales, NINO is found to drive Indian Monsoon and there is insignificant effect in the opposite direction.

Although the NAO is known to be a leading mode of winter climate variability over Europe^109–111^, the directionality or feedback in NAO related climate effects has been studied by a few causality analysis studies^9,112,113^. We investigate the NAO-European temperatures relationship on both monthly and daily time scales using winter only data. While PCCC indicates that NAO drives central European temperatures with no significant feedback on the longer monthly scale, on the daily scale it shows no significant causation in either direction. On the other hand, CCC and CMI, based on one dimensional time series, indicate a strong influence from NAO to Frankfurt daily mean temperatures. This result indicates that the NAO influence on European winter temperature on the daily scale can be explained as a simple time-delayed transfer of information between scalar time series in which no role is played by higher-dimensional patterns, potentially reflected in ordinal coding. Such an information transfer in the atmosphere is tied to the transfer of mass and energy as indicated in the study of climate networks by Hlinka et al.^114^. CMI and PCMI estimates can be considered to be reliable for this analysis as the time-series analyzed are long, close to 6000 time points.

CCC is free of the assumptions of linearity, requirement of long-term stationarity, extremely robust to missing samples, irregular sampling and short length data; and its combination with permutation patterns allows it to make reliable inferences for coupled systems with multiple variables. Thus, we can expect our analysis and inferences presented here on some highly-researched and long-debated climatic interactions to be highly robust and reliable. We also expect that the use of PCCC on other challenging datasets from climatology and other fields will be helpful to shed light on the causal linkages in considered systems.

## Methods

*Compression-complexity causality (CCC)* is defined as the change in the dynamical compression-complexity of time series *y* when $\Delta y$ is seen to be generated jointly by the dynamical evolution of both $y_{past}$ and $x_{past}$ as opposed to by the reality of the dynamical evolution of $y_{past}$ alone. $y_{past}, x_{past}$ are windows of a particular length *L* taken from contemporary time points of time series *y* and *x* respectively and $\Delta y$ is a window of length *w* following $y_{past}$^41^. Dynamical compression-complexity (CC) is estimated using the measure effort-to-compress (ETC)^115^ and given by:

3 $$\begin{aligned}&CC(\Delta y \vert y_{past} )=ETC(y_{past}+\Delta y)-ETC(y_{past} ), \end{aligned}$$

4 $$\begin{aligned}&CC(\Delta y \vert y_{past}, x_{past} ) = ETC(y_{past}+\Delta y, x_{past}+\Delta y) - ETC(x_{past},y_{past} ), \end{aligned}$$

Equation (3) computes the dynamical compression-complexity of $\Delta y$ as a dynamical evolution of $y_{past}$ alone. Equation (4) computes the dynamical compression-complexity of $\Delta y$ as a dynamical evolution of both $y_{past}$ and $x_{past}$. $CCC_{x_{past} \rightarrow y}$ is then estimated as:

5 $$\begin{aligned} CCC_{x_{past} \rightarrow \Delta y}= CC(\Delta y \vert y_{past} ) - CC(\Delta y \vert y_{past}, x_{past} ). \end{aligned}$$

Averaged CCC from *x* to *y* over the entire length of time series with the window $\Delta y$ being slided by a step-size of $\delta$ is estimated as:

6 $$\begin{aligned} \begin{aligned} CCC_{x \rightarrow y}&= {\overline{CCC}}_{x_{past} \rightarrow \Delta y} \\&= {\overline{CC}}(\Delta y \vert y_{past} ) - {\overline{CC}}(\Delta y \vert x_{past}, y_{past} ), \end{aligned} \end{aligned}$$

If ${\overline{CC}}(\Delta y \vert y_{past} ) \approx {\overline{CC}}(\Delta y \vert x_{past}, y_{past})$, there is no causality from *x* to *y*. Surrogate time series are generated for both *x* and *y* and the $CCC_{x \rightarrow y}$ values of the original and surrogate time series compared. If the CCC computed for original time series is statistically different from that of surrogate time series, we can infer the presence of causal relation from $x \rightarrow y$^42^. $CCC_{x \rightarrow y}$ can be both < or $> 0$ depending upon the *nature* or *quality* of the causal relationship^41^. The magnitude indicates the strength of causation.

Selection of parameters: $L,w,\delta$ and the number of bins, *B*, for symbolizing the time series using equidistant binning (ETC is applied to symbolic sequences) is done using parameter selection criteria given in the supplementary text of Ref.^41^.

*Permutation compression-complexity causality* is the causal inference technique proposed and implemented in this work. Given a pair of time series $x_1$ and $x_2$ from dynamical systems in which causation is to be checked from $x_1$ to $x_2$, we first embed the time series of the potential driver ($x_1$ here) in the following manner: $x_1(t), x_1(t+\eta ), x_1(t+2\eta ), \ldots x_1(t+(m-1)\eta )$, where $\eta$ is the time delay and *m* is the embedding dimension of $x_1$. $\eta$ is computed as the first minimum of auto mutual information function. The embedded time-series at each time-point is then symbolized using permutation or ordinal patterns binning. For example, if $m=3$, the embedding at time point *t* is given as ${\hat{x}}_1(t)=(x_1(t), x_1(t+\eta ), x_1(t+2\eta ))$. Symbols 0, 1, 2 are then used for labelling the pattern for ${\hat{x}}_1(t)$ at each time point by sorting the embedded values in ascending order, with 2 being used for the highest value and 0 for the lowest. If two or more values are exactly same in ${\hat{x}}_1(t)$, they are labelled differently depending on the order of their occurrence, where the same value takes a smaller symbol at its first (or earlier) occurrence. However, this may lead to two or more different embedded vectors having the same ordinal representation. For example, the embeddings, (3, 5, 5), (3, 3, 5) and (3, 3, 3), all have an ordinal representation of (0, 1, 2). This limits the total number of possible patterns at time *t* to $m!=3!$. Thus, ${\hat{x}}_1(t)$ is symbolized to a one dimensional sequence consisting of *m*! possible symbols or bins. *CCC* is then estimated from ${\hat{x}}_1(t)$ to $x_2(t)$, using Eq. (6) after symbolizing $x_2(t)$ using standard equidistant binning with *m*! bins. Thus,

7 $$\begin{aligned} PCCC_{x_1 \rightarrow x_2}=CCC_{{\hat{x}}_1 \rightarrow x_2}. \end{aligned}$$

Permutation binning is not done for the potential driver series as it was found from simulation experiments (Rössler data) that embedding the ‘cause’ alone works better for the CCC measure. Full dimensionality of the cause is necessary to predict the effect. Hence, embedding only the cause helps to recover the causal relationship. PCCC helps to take into account the multidimensional nature of the coupled systems. Parameter selection for PCCC is done in the same manner as for the case of CCC, using the symbolic sequences, ${\hat{x}}_1(t)$ and $x_2(t)$, for selection of the parameters. When PCCC is to be estimated from $x_2 \rightarrow x_1$, $x_2$ is embedded and $x_1$ remains as it is. Just like CCC, the PCCC measure can also take negative values.

*Conditional mutual information (CMI)* of the variables *X* and *Y* given the variable *Z* is a common information-theoretic functional used for the causality detection, and can be obtained as

8 $$\begin{aligned} I(X;Y|Z)=H(X|Z)+H(Y|Z)-H(X,Y|Z) \end{aligned}$$

where $H(X_1,X_2,...|Z)=H(X_1,X_2,...)-H(Z)$ is the conditional entropy, and the joint Shannon entropy $H(X_1,X_2,...)$ is defined as:

9 $$\begin{aligned} H(X_1,X_2,...)=-\sum _{x_1,x_2,...}{p(x_1,x_2,...)\log {p(x_1,x_2,...)}} \end{aligned}$$

where $p(x_1,x_2,...)=Pr[X_1=x_1,X_2=x_2,...]$ is the joint probability distribution function of the amplitude of variables $\{X_1,X_2,...\}$. In order to detect the coupling direction among two dynamical variables of *X* and *Y*, Paluš et al.^21^ used the conditional mutual information $I(X(t);Y(t+\tau )|Y(t))$, that captures the net information about the $\tau$-future of the process *Y* contained in the process *X*. As mentioned in the Introduction, to estimate other unknown variables, an *m*-dimensional state vector *X* can be reconstructed as $X(t)=\{x(t),x(t-\eta ),...,x(t-(m-1)\eta )\}$. Accordingly, CMI defined above can be represented by its reconstructed version for all variables of *X*(*t*), $Y(t+\tau )$ and *Y*(*t*). However, extensive numerical studies^22^ demonstrated that CMI in the form

10 $$\begin{aligned} I(X(t);Y(t+\tau )|Y(t),Y(t-\eta ),...,Y(t-(m-1)\eta )) \end{aligned}$$

is sufficient to infer direction of coupling among dynamical variables of *X*(*t*) and *Y*(*t*). In this respect, we use this measure to detect causality relationships in this article.

*Permutation conditional mutual information (PCMI)* can be obtained based on the permutation analysis described earlier in the PCCC definition. In this approach, all marginal, joint or conditional probability distribution functions of the amplitude of the variables are replaced by their symbolized versions, thus Eq. (9) should be replaced by

11 $$\begin{aligned} H({\hat{X}}_1,{\hat{X}}_2,...)=-\sum _{{\hat{x}}_1,{\hat{x}}_2,...}{p({\hat{x}}_1,{\hat{x}}_2,...)\log {p({\hat{x}}_1,{\hat{x}}_2,...)}} \end{aligned}$$

where $p({\hat{x}}_1,{\hat{x}}_2,...)=Pr[{\hat{X}}_1={\hat{x}}_1,{\hat{X}}_2={\hat{x}}_2,...]$ is the joint probability distribution function of the symbolized variables ${\hat{X}}_i(t)=\{X_i(t),X_i(t+\eta ),...,X_i(t+(m-1)\eta )\}$. By using Eqs. (8) and (11), permutation CMI can be obtained as $I({\hat{X}}(t);{\hat{Y}}(t+\tau )|{\hat{Y}}(t))$. Finally, one should replace $\tau$ with $\tau +(m-1)\eta$ in order to avoid any overlapping between the past and future of the symbolized variable ${\hat{Y}}$.

*Parameters of the methods* used were set as shown in Table 2 for different datasets.

**Table 2:** Parameters corresponding to each method, used for different datasets.

Dataset	Embedding	CCC	PCCC	CMI/ PCMI
Rössler	$\eta _{x_1}=5$, $\eta _{x_2}=5$, $m=3$	$L=300$, $w=30$, $\delta =30$, $B=8$	$L=25$, $w=15$, $\delta =20$	$\tau =20$
Millenial CO$_{2}$-T	$\eta _{CO_2}=11$, $\eta _{T}=16$, $m=3$	$L=60$, $w=15$, $\delta =20$, $B=4$	$L=60$, $w=30$, $\delta =20$	$\tau =1-30$
Kilo-year CO$_{2}$-T	$\eta _{CO_2}=24$, $\eta _{T}=8$, $m=3$	$L=60$, $w=15$, $\delta =20$, $B=4$	$L=30$, $w=15$, $\delta =20$	$\tau =1-30$
Kilo-year CH_4_-T	$\eta _{CH_4}=10$, $\eta _{T}=8$, $m=3$	$L=60$, $w=15$, $\delta =20$, $B=4$	$L=30$, $w=15$, $\delta =20$	$\tau =1-30$
Monthly CO$_{2}$-T	$\eta _{CO_2}=3$, $\eta _{T}=2$, $m=3$	$L=60$, $w=15$, $\delta =20$, $B=4$	$L=30$, $w=15$, $\delta =20$	$\tau =1-30$
Yearly ENSO-SASM	$\eta _{ENSO}=1$, $\eta _{SASM}=4$, $m=3$	$L=60$, $w=15$, $\delta =20$, $B=4$	$L=60$, $w=30$, $\delta =30$	$\tau =1-30$
Monthly NINO-India Monsoon	$\eta _{NINO}=10$, $\eta _{mon}=3$, $m=3$	$L=60$, $w=15$, $\delta =20$, $B=4$	$L=30$, $w=15$, $\delta =20$	$\tau =1-30$
Monthly NAO-T	$\eta _{NAO}=1$, $\eta _{T}=1$, $m=3$	$L=60$, $w=15$, $\delta =20$, $B=4$	$L=30$, $w=15$, $\delta =10$	$\tau =1-30$
Daily NAO-T	$\eta _{NAO}=15$, $\eta _{T}=15$, $m=3$	$L=40$, $w=15$, $\delta =20$, $B=4$	$L=30$, $w=15$, $\delta =20$	$\tau =1-30$

## Data Availability

The millenial scale CO$_{2}$ and temperature datasets are freely available at https://zenodo.org/record/4562996#.YiDbTN_ML3A. Kilo-year scale CO$_{2}$, CH$_{4}$ and temperature datasets are available as supplementary files for Ref.^64^ at https://agupubs.onlinelibrary.wiley.com/doi/full/10.1002/2015RG000482. Monthly CO$_{2}$ recordings are taken from the NOAA repository and are available at https://gml.noaa.gov/ccgg/trends/. Monthly Northern hemisphere temperature anomaly recordings are taken from the NOAA repository and are available at https://www.ncdc.noaa.gov/cag/global/time-series. The yearly El Niño/Southern Oscillation Index Reconstruction dataset is taken from the NOAA repository, https://www.ncei.noaa.gov/access/paleo-search/study/11194. The yearly South Asian Summer Monsoon Index Reconstruction dataset is taken from the NOAA repository, https://www.ncei.noaa.gov/access/paleo-search/study/17369. Monthly Niño 3.4 SST Index dataset is taken from the NOAA repository, available at https://psl.noaa.gov/gcos_wgsp/Timeseries/Nino34/. Monthly all India rainfall dataset is made available by the World Metereological Organization at http://climexp.knmi.nl/data/pALLIN.dat. Reconstructed monthly North Atlantic Oscillation Index is available at the NOAA repository, https://psl.noaa.gov/gcos_wgsp/Timeseries/RNAO/. Monthly Central European 500 Year Temperature Reconstructions are available at the NOAA repository, https://www.ncei.noaa.gov/access/metadata/landing-page/bin/iso?id=noaa-recon-9970. Daily North Atlantic Oscillation Index is available at the NOAA repository, https://www.cpc.ncep.noaa.gov/products/precip/CWlink/pna/nao.shtml. Daily Frankfurt air temperatures are made available by the ECA &D project at https://www.ecad.eu/dailydata/predefinedseries.php.

## Code availability

The computer codes used in this study are freely available at https://github.com/AditiKathpalia/PermutationCCC under the Apache 2.0 Open-source license.
